# The complete plastid genome of a marine microalgae *Cryptophyceae sp.* CCMP2293 (Cryptophyta)

**DOI:** 10.1080/23802359.2019.1623725

**Published:** 2019-07-10

**Authors:** Kuipeng Xu, Shunxin Hu, Xianghai Tang

**Affiliations:** College of Marine Life Sciences, Ocean University of China, Qingdao, China

**Keywords:** Cryptophyceae sp. CCMP2293, plastid genome, phylogenetic analysis

## Abstract

In this study, we present the complete plastid genome of *Cryptophyceae sp.* CCMP2293. The circular genome is 139,208 bp in length and contains 142 protein-coding genes (PCGs), 30 transfer RNA (tRNA) genes, 6 ribosome RNA (rRNA) genes, and 1 transfer-messenger RNA (tmRNA) gene. The overall nucleotide composition is: 33.6% A, 32.5% T, 16.8% C, and 17.1% G with a total A + T content of 66.1%. The phylogenetic tree was constructed to explore the taxonomic status of *Cryptophyceae sp.* CCMP2293, which is closely related to *G. theta* and *R. salina*.

Cryptophyte algae are an evolutionarily significant group which inhabits marine, brackish, and freshwater environments (Edwards et al. 2000; Shalchian-Tabrizi et al. 2008). Cryptophytes have strong adaptability to light and temperature and they become the dominant species in both winter and summer. Phototrophs of cryptophytes contain plastids with chlorophyll-a and -c, as well as phycobilins, as accessary pigments (Kim et al. 2018). There are four genomes in cryptophyte cells: host-derived nuclear and mitochondrial genomes, and plastid and nucleomorph genomes of endosymbiotic origin (Douglas et al. 1991). However, relatively little is known about plastid genome information of cryptophytes.

Here, we reported and characterized the complete *Cryptophyceae sp.* CCMP2293 plastid genome (accession number: MK798155). The single specimen was provided by the Culture Collection of Marine at the Ocean University of China in Qingdao (OUC-2013060210). Illumina paired-end DNA library was prepared and sequenced using HiSeq 2500 Sequencing System (Gene Denovo Laboratory). The pre-processed sequences were assembled using NOVOPlasty (Dierckxsens et al. 2017). The organellar genomes were annotated using ORF-finder (http://www.ncbi.nlm.nih.gov/projects/gorf/) and aligned via BLASTX and BLASTN searches at NCBI (http://blast.ncbi.nlm.nih.gov/). The tRNAs were identified using the tRNAscan-SE 1.21 web server (http://lowelab.ucsc.edu/tRNAscan-SE/), and the rRNAs were identified using the RNAmmer 1.2 server (http://www.cbs.dtu.dk/services/RNAmmer/). The complete plastid genome of *Cryptophyceae sp.* CCMP2293 is 139,208 bp in length and contains 142 protein-coding genes (PCGs), 30 transfer RNA (tRNA) genes, 6 ribosome RNA (rRNA) genes and 1 transfer-messenger RNA (tmRNA). There are 4 function-unknown open reading frames. The overall nucleotide composition is: 33.6% A, 32.5% T, 16.8% C, and 17.1% G, with a total A + T content of 66.1%.

To elucidate the phylogenetic position of *Cryptophyceae sp.* CCMP2293, the phylogenetic tree was constructed with 6 published complete plastid genomes obtained from the Genbank, where *Costaria costata* served as outgroup. 56 concatenated protein-coding amino acid sequences were aligned using the program MAFFT (Katoh et al. 2005). Neighbour joining (NJ) phylogenetic tree was created using MEGA7 with 1000 bootstrap replicate (Kumar et al. 2016). The six sampled Cryptophyta taxa clustered together and robustly resolved. Phylogenetic analysis showed that *Cryptophyceae sp.* CCMP2293 clustered with *G. theta* and *R. salina* (Figure 1). The determination of the complete plastid genome sequences provided new molecular data to illuminate the Cryptophyta evolution.

**Figure 1.**
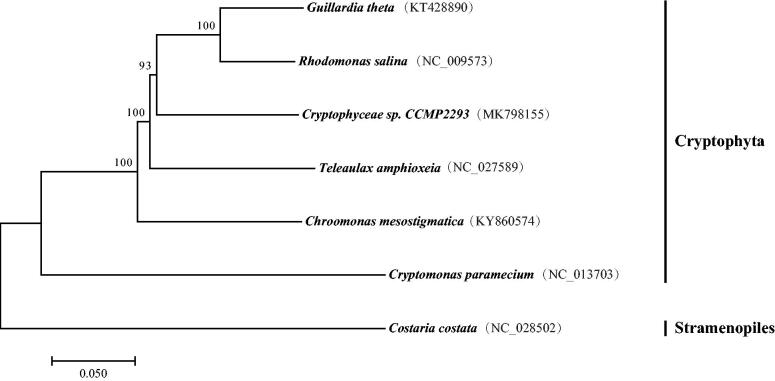
Neighbor-joining phylogenetic tree of the *Cryptophyceae sp.* CCMP2293 and six other species based on the concatenated sequences of 56 protein-coding genes. Numbers on nodes indicate bootstrap support value, based on 1000 replicates. The genbank accession numbers were in brackets.
